# Exploring Gastrodin Against Aging-Related Genes in Alzheimer’s Disease by Integrated Bioinformatics Analysis and Machine Learning

**DOI:** 10.3390/ijms26189097

**Published:** 2025-09-18

**Authors:** Lipeng Zhou, Xinying Chen, Shuang Liang, Jiulong Yan, Lianhu Sun, Yaping Li, Xingliang Chen, Zhirong Sun

**Affiliations:** 1 School of Chinese Materia Medica, Beijing University of Chinese Medicine, Beijing 102488, China; 20240941476@bucm.edu.cn (L.Z.); 20240935179@bucm.edu.cn (X.C.); 20250941552@bucm.edu.cn (S.L.); 20230932013@bucm.edu.cn (J.Y.); 20240935178@bucm.edu.cn (Y.L.); 2 Longnan Agricultural Science Research Institute, Longnan 746000, China; sun2265103@126.com; 3 Chinese Academy of Forestry, Beijing 100091, China

**Keywords:** gastrodin, Alzheimer’s disease, aging, machine learning, molecular dynamics simulation

## Abstract

Gastrodin is the main active ingredient of *Gastrodia elata* Blume, known for its prominent neuroprotective effects, especially in Alzheimer’s disease. Meanwhile, aging is a critical risk factor in age-related diseases, including AD. Now, the underlying mechanisms of gastrodin against aging-related genes in Alzheimer’s disease remain unclear. Our study aimed to identify and validate the molecular mechanisms of gastrodin on aging-related genes in Alzheimer’s disease. Firstly, we analyzed gene expression datasets from GEO in NCBI, and weighted gene co-expression network analysis (WGCNA) was used to identify the intersected genes with differentially expressed genes (DEGs) and aging genes. Gene Ontology (GO) and Kyoto Encyclopedia of Genes and Genomes (KEGG) functional enrichment analyses and protein–protein interaction (PPI) network were performed to analyze and evaluate the intersected genes to screen key genes. Subsequently, we used machine learning techniques to screen hub genes and subcellular localization to confirm the reliability of the hub genes. Finally, molecular docking and molecular dynamics simulation were combined to explore the binding interactions between core targets and gastrodin. We identified 29 intersecting genes among DEGs of AD, key module genes of AD, and aging genes. Next, nine common hub genes were identified by four algorithms of the cytoHubba plug-in. Four hub genes, *GFAP*, *NPY*, *SNAP25*, and *SST*, were found as possibly hub aging-related genes in Alzheimer’s disease from machine algorithms by adopting the random forest, LASSO, SVM, and Boruta models. Among them, *GFAP* showed marked upregulation, while *NPY*, *SNAP25*, and *SST* exhibited significant downregulation (*p* < 0.05). Finally, molecular docking and molecular dynamics simulation exhibited that gastrodin has an excellent affinity in docking with SNAP25. This study demonstrates that SNAP25 can be considered a key aging-related gene in AD, and gastrodin could treat AD by targeting specific genes and signaling pathways. These findings provide critical insights for the clinical application of gastrodin.

## 1. Introduction

Alzheimer’s disease (AD) is a progressive neurodegenerative disorder that most commonly affects people over 65 years, profoundly impairing cognitive functions and daily living activities of patients [1,2]. The disease is characterized by distinct neuropathological hallmarks, including extracellular accumulation of β-amyloid peptide-containing plaques (senile plaques) in cerebral cortical regions of AD patients [3], intracellular aggregation of hyperphosphorylated Tau proteins resulting in neurofibrillary tangles [4], and significant synaptic loss [5], and neuronal degeneration [6]. Meanwhile, aging is recognized as the key risk factor for Alzheimer’s disease, which imposes a heavy economic burden on both families and society [7]. It is well known that aging is closely related to the formation of neurodegenerative diseases [8]. In the pathogenesis of neurodegenerative diseases such as Alzheimer’s disease and Parkinson’s disease, there are pathological and physiological changes, such as the accumulation of misfolded proteins [2], mitochondrial dysfunction [9], and chronic neuroinflammation [10]. At the same time as these pathological and physiological changes occur, there is a close association with aging. Therefore, the novel aging-related pathological genes of AD are considered key points for the early diagnosis and the therapeutic development of AD.

*Gastrodia elata* Blume is well-known as a healthy food with rich nutritional value and tonic effect, which is widely used in clinical practice for the treatment of neurological disorders and age-related diseases, such as Alzheimer’s disease, Parkinson’s disease, and depressive disorders [11]. In addition to the neuroprotective and anti-aging qualities of the extract of *G. elata* in vitro and in vivo, the monomeric components from *G. elata* have also been shown to have strong neuroprotective and anti-aging benefits. Among these monomeric components, gastrodin is the main bioactive component in protecting against neuronal damage and enhancing cognitive function in animal models of Alzheimer’s disease, which is worth investing more effort in the research of neuroprotection [12]. Recently, our team investigated the mechanism of gastrodin intervention in AD and found that gastrodin could inhibit the ubiquitination of P-gp by binding to FBXO15, resulting in increased expression and transport function of P-gp protein [13]. This reminds us that gene expression analysis is becoming important in biological research, and targeted biomarkers could be a key point of gastrodin in the early prevention, treatment, and prognosis of AD.

**Figure 1:**
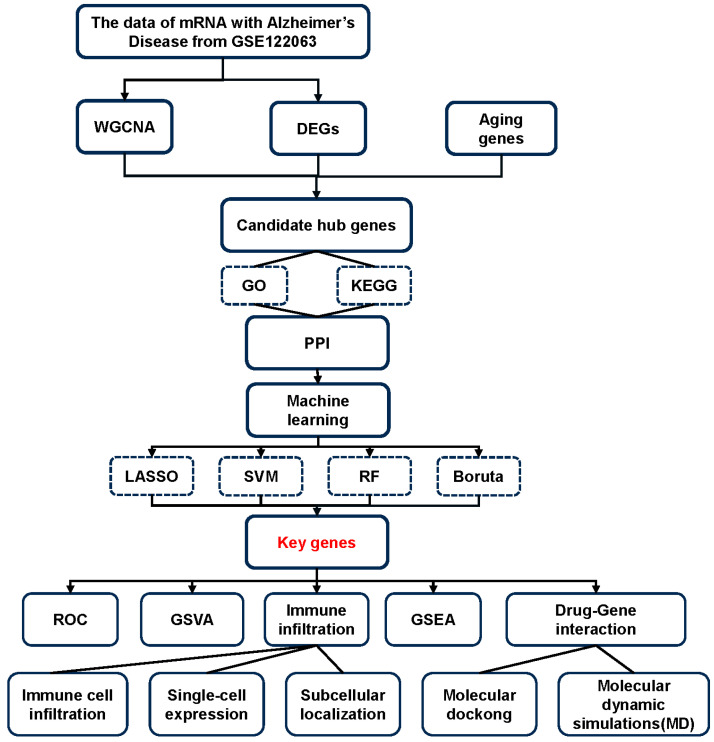
The flow chart of this research.

In this study, we obtained a microarray dataset of normal and AD temporal cortex samples from the Gene Expression Omnibus (GEO) database in NCBI. Through comprehensive analysis using weighted gene co-expression network analysis (WGCNA), differentially expressed genes (DEGs) and aging genes, we systematically screened samples from both healthy controls and AD patients to identify potential aging-related biomarkers in AD. To systematically identify key genes from the intersected genes, we conducted comprehensive functional enrichment analyses including Gene Ontology (GO) terms and Kyoto Encyclopedia of Genes and Genomes (KEGG) pathways, complemented by protein–protein interaction (PPI) network analysis. To further refine our analysis, machine learning algorithms were used to identify hub genes, which were subsequently validated in an independent dataset. At the same time, subcellular localization was used to validate the biological relevance and reliability of these findings. Finally, molecular docking and molecular dynamics simulations were combined to predict the binding interactions between core targets, providing a reference for generating new and effective treatments for AD. Meanwhile, the flow chart of this study is illustrated in Figure 1.

## 2. Results

### 2.1. Construction of Weighted Gene Co-Expression Networks

**Figure 2:**
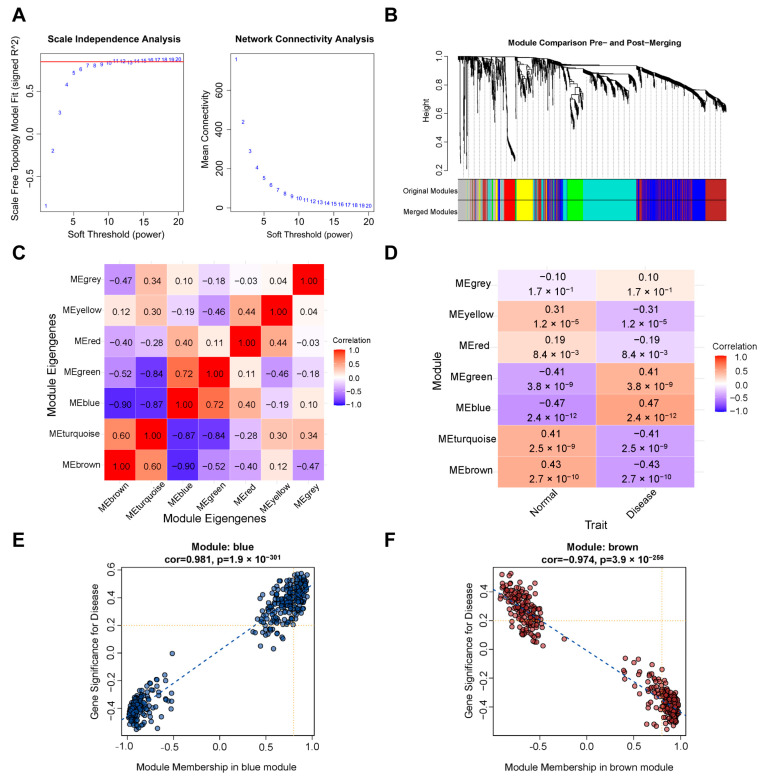
The WGCNA of GSE132903. (**A**) The soft threshold power of WGCNA. (**B**) Gene system clustering tree and corresponding modules drawn based on TOM. (**C**) Heatmap of the association among modules. (**D**) Heatmap of module–trait relationships. (**E**) Correlation chart between gene members of the blue module and gene significance. (**F**) Correlation chart between gene members of the brown module and gene significance.

We conducted WGCNA to analyze the dataset GSE132903 to screen the key gene modules closely associated with AD. A scale-free network was employed to identify that the soft threshold power β was set to 11 and R2 = 0.9 as a criterion (Figure 2A). As depicted in Figure 2B, a total of seven gene modules (gray, yellow, red, green, blue, turquoise, and brown ) were obtained by the dynamic tree cut algorithm. As a result, their associations were shown in Figure 2C. The MEbrown module (cor = −0.43; *p* = 2.7 × 10^−10^) and the MEblue module (cor = 0.47; *p* = 2.4 × 10^−12^) had the strongest negative and positive connections with AD, respectively, presented with heat maps in Figure 2D. Among them, the brown module was identified as 397 module genes, and the blue module was analyzed as 423 module genes (Supplementary Table S2).

### 2.2. Identification of Differentially Expressed Genes and Aging-Related Differential Expression Genes

**Figure 3:**
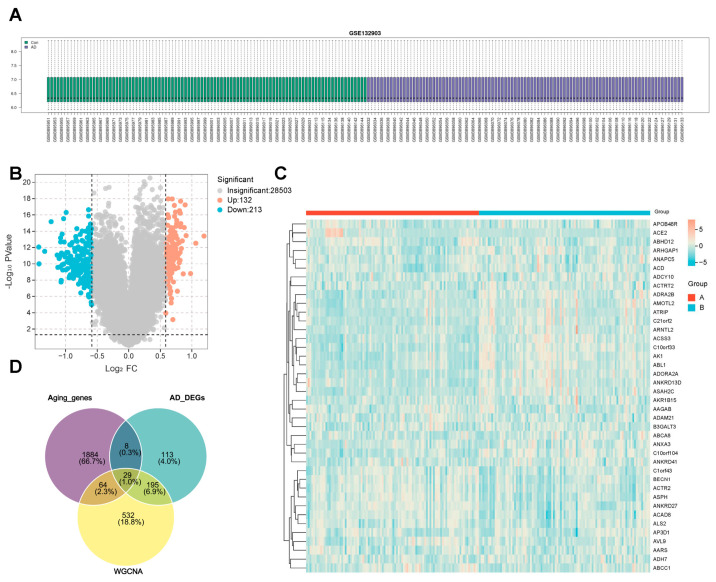
Identifying the DEGs of AD. (**A**) Data standardization of GSE132903. (**B**) The volcano plot of DEGs of AD. (**C**) The heat map of the top 40 DEGs of AD. (**D**) The Venn diagram of the DEGs of AD DEGs, WGCNA, and aging genes.

Differentially expressed genes (DEGs) between normal and AD samples were identified through the “limma” package 3.64.1, and the expression profile datasets were normalized (Figure 3A). A total of 345 DEGs (Supplementary Table S3) were selected and detected, and the volcano plots were generated (Figure 3B), with 213 genes down-regulated and 132 genes up-regulated. The top 20 up-regulated and top 20 down-regulated DEGs are shown in the heatmap (Figure 3C). In Figure 3D and Table S2, we recognized 29 aging-related differential expression genes via integrated analysis of the DEGs, genes from key modules (WGCNA), and aging genes.

### 2.3. Functional Enrichment Analysis of Aging-Related Differential Expression Genes

**Figure 4:**
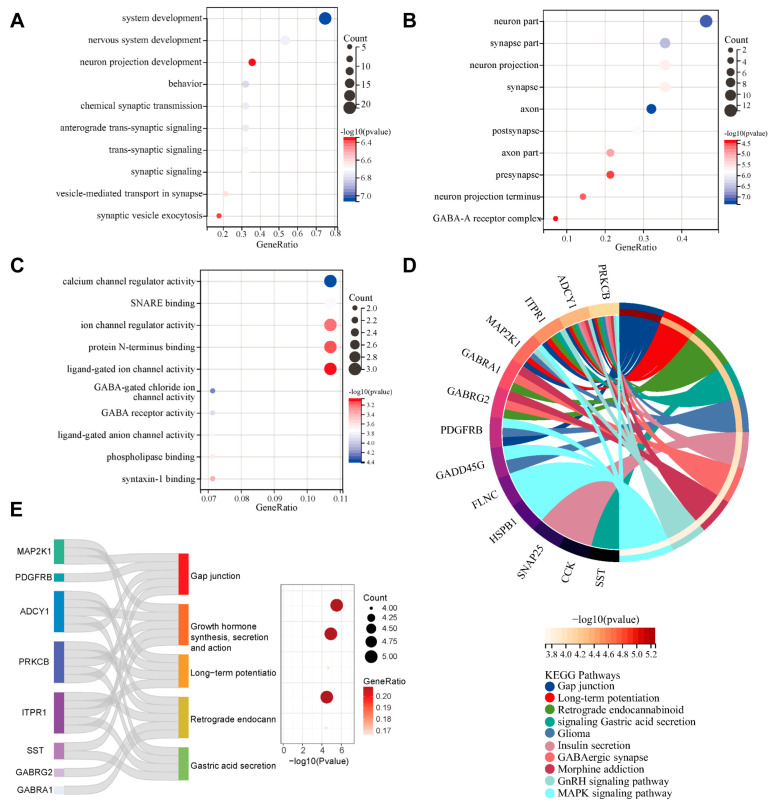
The items of the function enrichment analyses of the aging-related differential expression genes. (**A**) GO biological processes (BP); (**B**) GO cellular components (CC); (**C**) GO molecular functions (MF); (**D**) KEGG pathways; (**E**) Sankey diagram of the top-ranked KEGG channel.

We performed Functional enrichment analysis on the above aging-related differential expression genes (ARDEGs) to reveal comprehensive molecular insights into Biological Processes (BPs), cellular components (CCs), and molecular functions (MFs) of Gene Ontology (GO) analysis, and the KEGG pathway. For the Biological Process category, in terms of biological processes, the ARDEGs were associated with system development, nervous system development, and behavior (Figure 4A). Figure 4B demonstrates the cellular components, indicating that ARDEGs were enriched in the neuron part, the synapse part, and the neuron projection, etc. As depicted in Figure 4C, in terms of molecular functions, ARDEGs were mainly involved in calcium channel regulator activity, SNARE binding, and ion channel regulator activity, etc. As shown in Figure 4D,E, KEGG pathway enrichment analysis demonstrated that the main ARDEGs were predominantly associated with gap junction, growth hormone synthesis, secretion and action, long-term potentiation, retrograde endocannabinoid signaling, and gastric acid secretion.

### 2.4. PPI Network and Hub Genes Analysis

**Figure 5:**
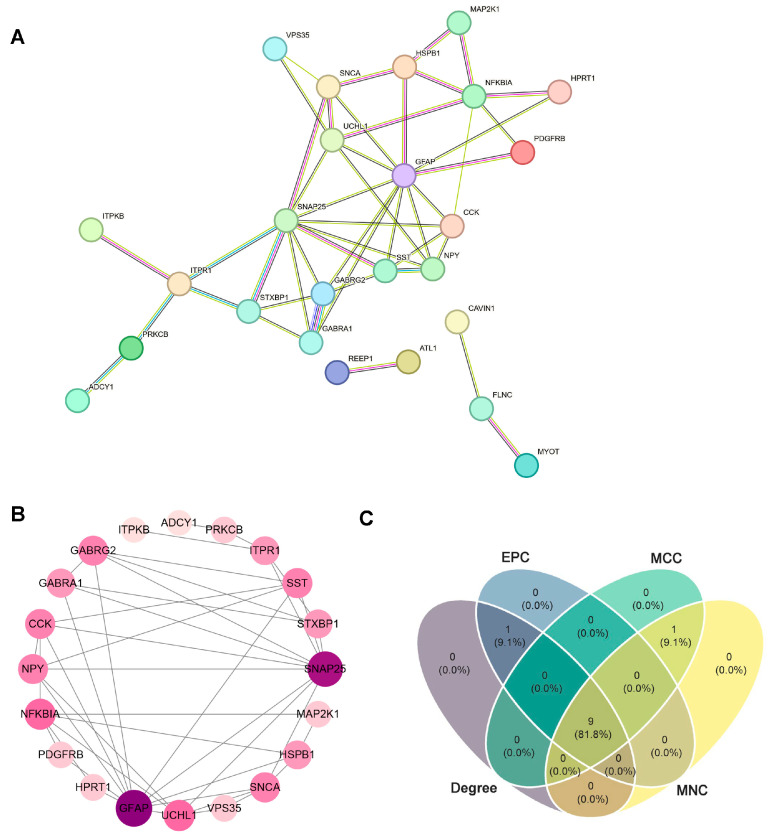
Protein–protein interaction (PPI) network and hub genes analysis. (**A**) The PPI network of the aging-related differential expression genes. (**B**) The first module of the PPI network. (**C**) Nine common hub genes were identified by four algorithms of cytoHubba plug-in. DEGs, differentially expressed genes.

The STRING tool 12.0 was used to construct and visualize a PPI network based on the ARDEGs to explain the interaction relationships between proteins (Figure 5A). The network has 20 nodes and 20 edges. To identify the most densely connected module in the network, we use the CytoHubba plugin and the MCODE plugin to identify the top 20 hub genes (Figure 5B). Through the EPC, MCC, MNC, and Degrer topological algorithm, nine common hub genes were obtained, including *GFAP*, *SNAP25*, *UCHL1*, *CCK*, *SST*, *NPY*, *GABRG2*, *SNCA*, and *GABRA1* (Figure 5C and Table 1).

### 2.5. Feature Gene Selection Based on Various Machine Learning Algorithms

**Figure 6:**
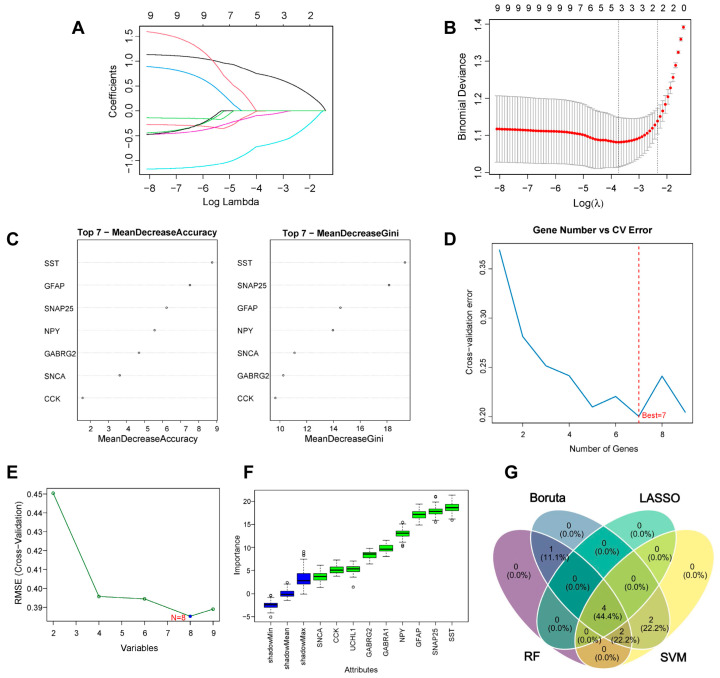
Identification of the key genes. (**A**,**B**) Four key genes were identified from hub genes by machine learning LASSO regression. (**C**,**D**) Seven key genes were also identified from hub genes by the random Forest (RF) algorithm. (**E**) Eight key genes were identified from hub genes by support vector machines (SVM). (**F**) Five key genes were identified from hub genes by Boruta. (**G**) Four key genes were identified by overlapping: *GFAP*, *NPY*, *SNAP25*, and *SST*.

Four machine learning algorithms (SVM, LASSO, RF, and Boruta) were employed to screen the above common hub genes. Firstly, through LASSO regression analysis, four feature genes that exhibited the smallest prediction errors were identified (Figure 6A,B). In contrast, we identified the optimal seven feature genes through the RF algorithm (Figure 6C,D). Similarly, the SVM regression algorithm subsequently refined this selection, identifying eight core targets with optimal predictive performance (Figure 6E). Based on the Boruta algorithm, the top 1 feature genes of relative importance were selected (Figure 6F). To further accurately screen hub genes, the intersection of the four algorithms was taken to obtain four core targets utilizing Venn diagrams (Figure 6G), including *GFAP*, *NPY*, *SNAP25*, and *SST*.

### 2.6. Identification and Validation of the Key Genes

**Figure 7:**
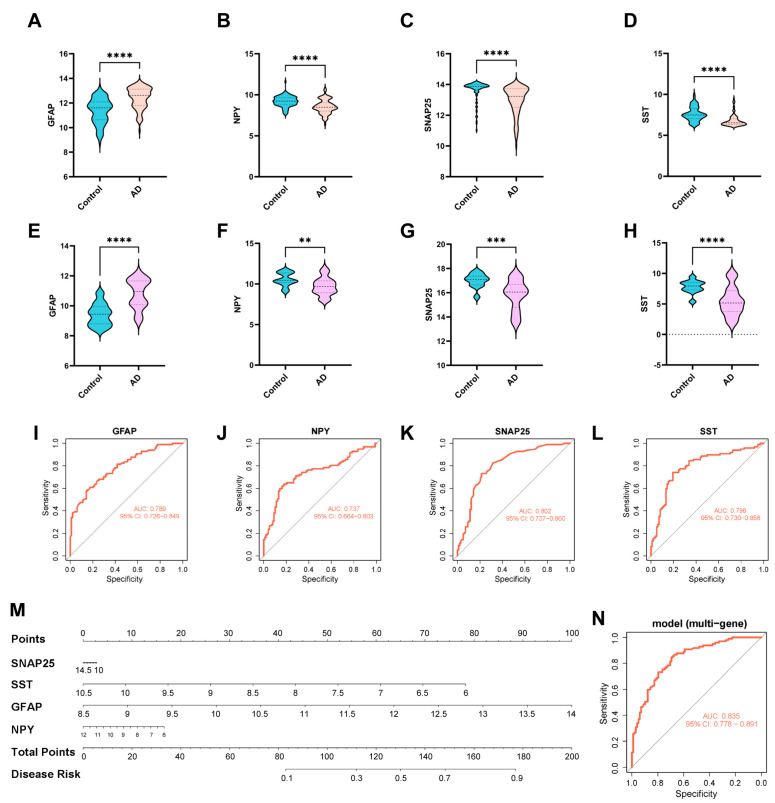
Expression of signature genes. (**A**–**D**) Expression of signature genes between AD patients and healthy controls in the training dataset file (Number: GSE132903). (**E**–**H**) Expression of signature genes between AD patients and healthy controls in the test dataset file (Number: GSE122063). (**I**–**L**,**N**) ROC shows the diagnostic performance of the signature genes. (**M**) The visible nomogram for diagnosing AD. **, *p* < 0.01. ***, *p* < 0.001. ****, *p* < 0.0001.

Compared with the normal group, the expression profiles of the four core targets in AD patients showed significant differential expression of all key genes. Among them, we found that the expression of GFAP showed an upregulating trend in the model group (Figure 7A); conversely, the expression of *NPY*, *SNAP25*, and *SST* showed the opposite trend (Figure 7B–D). In addition, the trends of these genes were verified in the test dataset GSE122063 (Figure 7E–H), consistent with the trend in the training dataset file (Number: GSE132903). Subsequently, the model was tested using ROC curves, which showed AUC values for *GFAP*, *NPY*, *SNAP25*, and *SST* as 0.789, 0.737, 0.802, and 0.796, respectively, indicating excellent diagnostic performance for AD (Figure 7I–L). Furthermore, as shown in Figure 7M, we establish a nomogram to determine the likelihood of developing aging-related Alzheimer’s disease based on the four core targets, achieving an AUC value of 0.853 (Figure 7N).

### 2.7. GSVA

**Figure 8:**
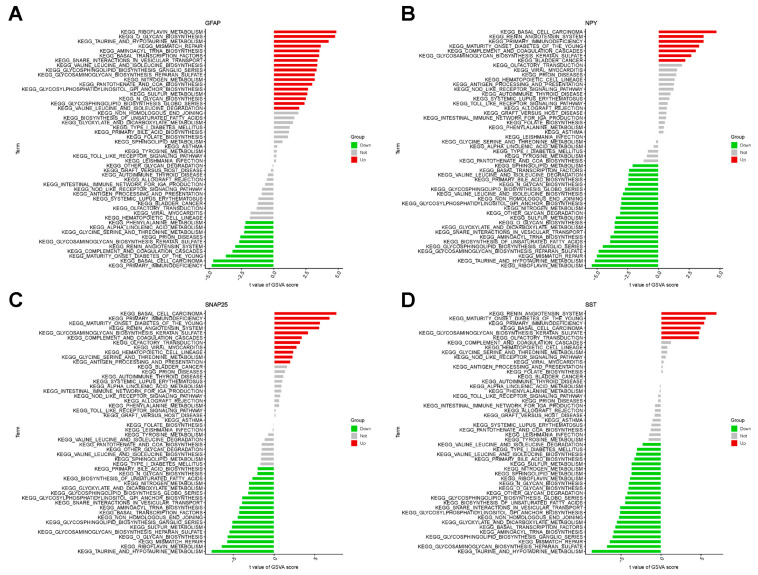
GSVA results for the four ERRGs. (**A**) *GFAP*, (**B**) *NPY*, (**C**) *SNAP25*, and (**D**) *SST*-associated signaling pathways. Red bars: pathways enriched in the high-expression group; green bars: pathways enriched in the low-expression group; gray bars: non-significant pathways. Bar plots show t-values of GSVA scores.

Aside from identification and validation of the key genes, expression analysis of the above four key genes was evaluated in the GSEA, which revealed significant correlations and key signaling pathways (Figure 8A–D). High *GFAP* expression notably upregulated pathways related to riboflavin metabolism, O-glycan biosynthesis, and taurine and hypotaurine metabolism, while downregulating primary immunodeficiency, basal cell carcinoma, and maturity-onset diabetes of the young. High expression of *NPY* was significantly associated with basal cell carcinoma, renin angiotensin system and primary immunodeficiency, while showing negative correlations with, while showing negative correlations with riboflavin metabolism, taurine and hypotaurine metabolism, and mismatch repair. For *SNAP25*, high expression was significantly associated with basal cell carcinoma, primary immunodeficiency, and maturity-onset diabetes of the young. Similarly, high expression of *SST* upregulated the renin angiotensin system, maturity-onset diabetes of the young, and primary immunodeficiency, while downregulating taurine and hypotaurine metabolism, glycosaminoglycan biosynthesis, heparan sulfate, and mismatch repair.

### 2.8. Immune Cell Infiltration Analysis

**Figure 9:**
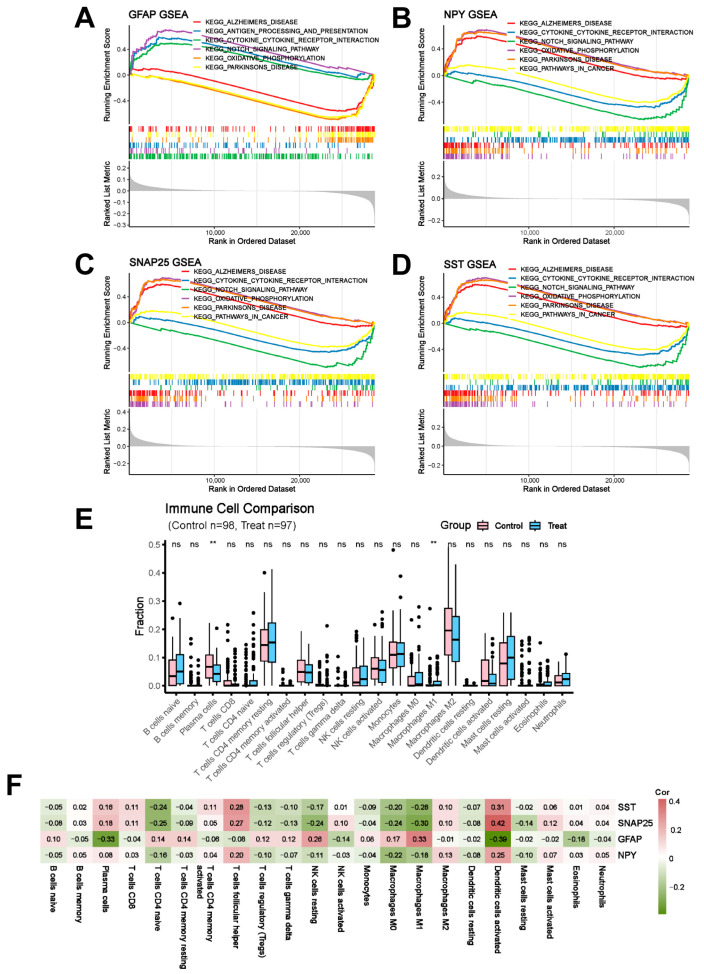
GSEA and immune infiltration analysis of genes characteristic of Fanconi anemia. (**A**) GSEA of *GFAP* in AD. (**B**) GSEA of *NPY* in AD. (**C**) GSEA of *SNAP25* in AD. (**D**) GSEA of *SST* in AD. (**E**) AD and immune cell infiltration in a healthy population. (**F**) Association between characterized genes and significantly different immune cell infiltration. “ns” indicates *p* ≥ 0.01. ** *p* < 0.001.

To elucidate the regulatory patterns of signaling pathways associated with core genetic factors, Gene Set Enrichment Analysis (GSEA) was systematically performed on *GFAP*, *NPY*, *SNAP25*, and *SST* (Figure 9A–D). GSEA of *GFAP* revealed that Alzheimer’s disease, anting processing and presentation, cytokine–cytokine receptor interaction showed an overall upregulation trend; while notch signaling pathway, oxidative phosphorylation, and Parkinson’s disease were identified showing an overall downregulation trend. GSEA of *NPY* and *SST* revealed that Alzheimer’s disease, the cytokine–cytokine receptor interaction, and the notch signaling pathway showed an overall upregulation trend. In contrast, oxidative phosphorylation, Parkinson’s disease, and pathways in cancer showed an overall downregulation trend. GSEA of *SNAP25* revealed that Alzheimer’s disease, cytokine–cytokine receptor interaction, and notch signaling pathway were identified, showing an overall upregulation trend. In contrast, oxidative phosphorylation, Parkinson’s disease, and pathways in cancer showed an overall downregulation trend.

Subsequently, we explored the composition of 22 immune cell types in normal and AD samples using the CIBERSORT algorithm. As a result, the proportions of plasma cells showed marked upregulation in the normal samples compared with the AD samples (Supplementary Table S4). Furthermore, the proportions of macrophages M1 were notably upregulated between normal and AD samples (Figure 9E). We analyzed the relationships between the four core targets and immune cell infiltration (Figure 9F). A negative correlation was seen between the expression levels of *GFAP*, *NPY*, *SNAP25*, and *SST* and the infiltration scores of dendritic cells resting and mast cells resting.

### 2.9. Single-Cell Analysis and Subcellular Localization of Hub Genes

**Figure 10:**
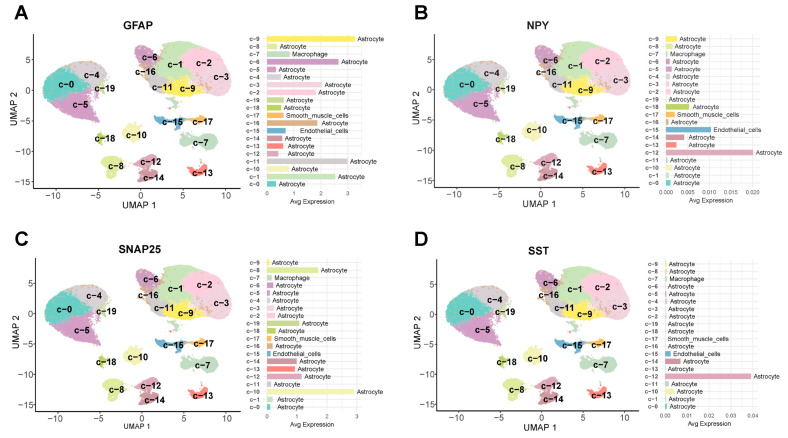
Single-cell expression analysis in temporal cortex tissues. (**A**) Expression distribution of GFAP protein in the temporal cortex. (**B**) Expression distribution of NPY protein in the temporal cortex. (**C**) Expression distribution of SNAP25 protein in the temporal cortex. (**D**) Expression distribution of SST protein in the temporal cortex.

Since the GSE167490 dataset was obtained from the temporal cortex of AD patients, single-cell transcriptomics was utilized to thoroughly investigate the gene expression profiles of specific cellular components within the temporal cortex tissues and their interactions. The results showed 20 regions in the temporal cortex tissues, including C0-astrocyte cells, C1-astrocyte cells, C2-astrocyte cells, C3-astrocyte cells, C4-astrocyte cells, C5-astrocyte cells, C6-astrocyte cells, C7-macrophage cells, C8-astrocyte cells, C9-astrocyte cells, C10-astrocyte cells, C11-astrocyte cells, C12-astrocyte cells, C13-astrocyte cells, C14-astrocyte cells, C15-endothelial cells, C16-astrocyte cells, C17-smooth muscle cells, C18-astrocyte cells and C19-astrocyte cells. Among them, GFAP was predominantly expressed in C9-astrocyte cells and C11-astrocyte cells (Figure 10A). However, NPY was expressed in the other regions. Particularly, it was highly expressed in C12-astrocyte cells and C15-endothelial cells (Figure 10B). Meanwhile, the results showed that SST was expressed in C12-astrocyte cells (Figure 10C) and C14-astrocyte cells, and SNAP25 was expressed in C8-astrocyte cells and C10-astrocyte cells (Figure 10D).

**Figure 11:**
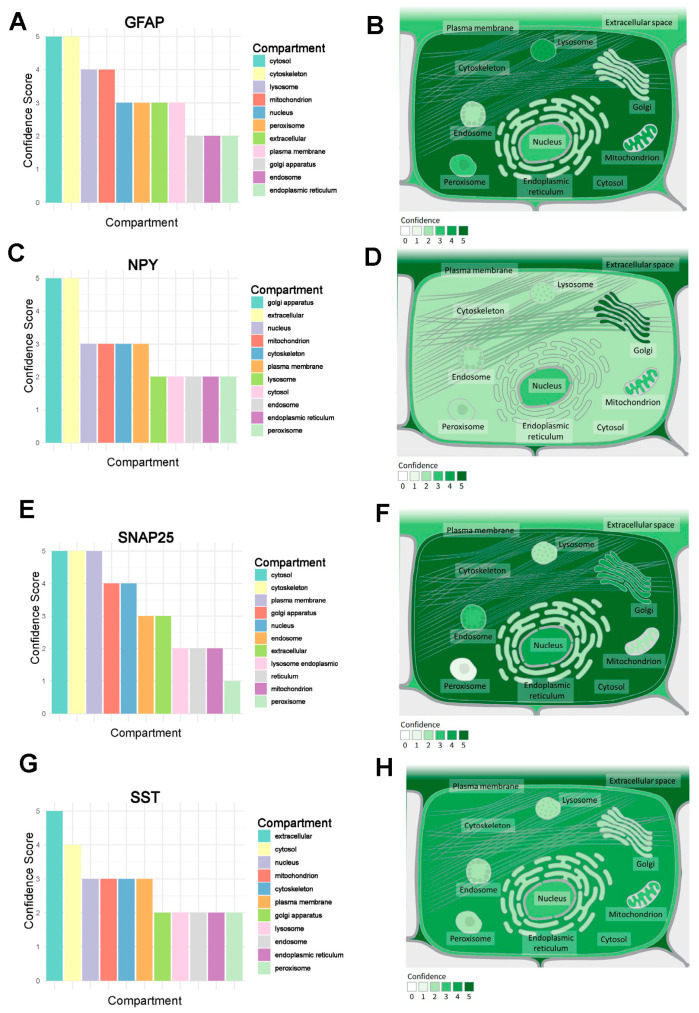
Subcellular localization of signature genes. (**A**,**B**) *GFAP* subcellular localization. (**C**,**D**) *NPY* subcellular localization. (**E**,**F**) *SNAP25* subcellular localization. (**G**,**H**) *SST* subcellular localization.

The results showed that *GFAP* was mainly distributed in the cytosol and cytoskeleton (Figure 11A,B) and *NPY* was distributed primarily on the Golgi apparatus and outside the cell (Figure 11C,D). Furthermore, *SST* was mainly distributed outside the cell (Figure 11E,F), and *SNAP25* was mainly distributed in the cytosol, cytoskeleton, and plasma membrane (Figure 11G,H).

### 2.10. Prediction of Gastrodin for Aging-Related Genes in AD

**Figure 12:**
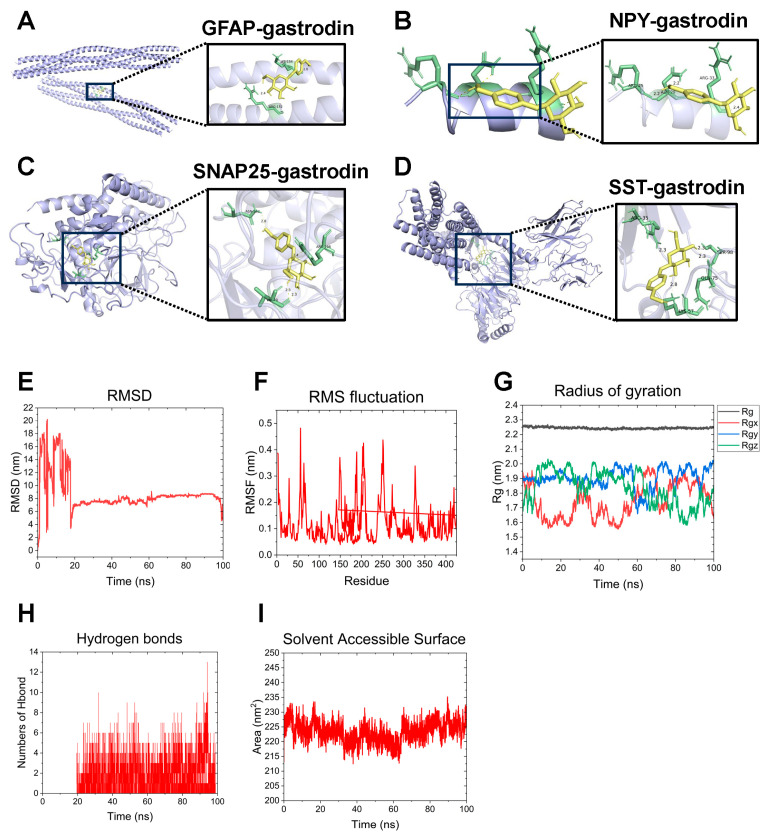
Molecular docking analysis of gastrodin with signature genes. (**A**–**D**) Binding interactions between gastrodin and GFAP (**A**), NPY (**B**), SNAP25 (**C**), and SST (**D**). (**E**) The fluctuation plot of the SNAP25–gastrodin complex’s RMSD values. (**F**) The fluctuation plot of SNAP25–gastrodin complex’s RMSF values. (**G**) Compactness of the protein according to Rg. (**H**) Hydrogen bond number of SNAP25–gastrodin complex. (**I**) The fluctuation plot of the SNAP25–gastrodin complex’s SASA values.

Based on the crystal structures of GFAP, NPY, SNAP25, and SST proteins obtained from the Protein Data Bank, along with the molecular structure of gastrodin from the PubChem database, we performed molecular docking of gastrodin with these targets. The results demonstrated that gastrodin displayed strong binding affinities with all four targets: GFAP, NPY, SNAP25, and SST, showing binding energies of –4.8, –4.9, –7.5, and –5.9 kcal/mol, respectively (Figure 12A–D). Notably, the lowest binding energy was observed between gastrodin and SNAP25 (–7.5 kcal/mol), indicating the strongest binding affinity of gastrodin for SNAP25 among the targets. For SNAP25, as shown in Figure 12C, gastrodin could form a hydrogen bond with ASN-362 (length = 2.8); a hydrophobic interaction bond (length = 2.3) and a hydrogen bond (length = 2.3) with VAL-70; a hydrogen bond with ARG-198 (length = 2.4). The bond lengths of the hydrogen bonds in Figure 12C (around 2.3) are consistent with the physical and chemical properties of hydrogen bonds, indicating that these interactions are stable within the binding pocket and play a crucial role in constraining the binding mode between the ligand and the target protein. The hydrophobic interaction between the aromatic ring in the figure and VAL-70 not only enhances the affinity of the ligand to the target protein but may also be involved in shape complementarity (such as the geometric matching between the aromatic ring and the hydrophobic pocket), further stabilizing the binding conformation [14]. Therefore, when SNAP25 acts as the target protein and gastrodin acts as the ligand, these interactions, including the hydrogen bonding and hydrophobic interactions, jointly determine the binding conformation of the ligand [15].

Next, we conducted molecular dynamics simulations of the gastrodin-SNAP25 complex to investigate the stability of the protein–ligand interactions further. Meanwhile, the molecular dynamics results are averages of replicate simulations. We utilized the root mean square deviation (RMSD) to assess whether the simulated system reached a stable state. An RMSD value within 1 nm indicates the relative stability of the SNAP25–gastrodin interaction in a physiological environment (Figure 12E). RMSF analysis showed fewer fluctuations in the gastrodin–SNAP25 complex, and the reduced surface area suggested that the complex became more tightly folded or bound during the simulation (Figure 12F). We also analyzed the radius of gyration (Rg) to evaluate the compactness of the receptor-ligand binding. The Rg value for the gastrodin–SNAP25 complex stabilized from approximately 2.2 nm to around 2.3 nm (Figure 12G). Furthermore, the solvent-accessible surface area (SASA) for the gastrodin–SNAP25 complex demonstrated stability, decreasing from 230 nm^2^ to 215 nm^2^ and reaching equilibrium at 60 ns (Figure 12H). Additionally, the number of hydrogen bonds in the gastrodin–SNAP25 complex reflected the strength of the protein–ligand interaction, showing stable hydrogen bond density and strength throughout the simulation (Figure 12I).

## 3. Discussion

With the intensification of the global aging problem, the number of people suffering from Alzheimer’s disease (AD) is constantly increasing, and it has become the third most fatal disease among the elderly, after cardiovascular and cerebrovascular diseases and malignant tumors [16]. The declarative and non-declarative memories of patients with AD are both affected. The patient exhibits progressive deterioration in cognitive function, manifesting as impaired comprehension and reasoning abilities, often accompanied by linguistic deficits [17]. Additionally, the condition frequently presents comorbid psychiatric symptoms, including depressive episodes, anxiety disorders, and persistent sleep disturbances. With the advancement of the neurodegenerative process, patients exhibit progressive and severe impairment in executive functioning [18]. This cognitive decline manifests as a gradual loss of instrumental activities of daily living (IADLs), ultimately resulting in complete functional dependence. Therefore, this study aims to identify the key aging genes associated with Alzheimer’s disease, in order to provide potential therapeutic strategies or optimized combination treatments to improve the outcomes for AD patients.

*Gastrodia elata* Blume (commonly known as Tianma) is a plant with dual medicinal and edible properties [19]. It is mainly used to treat diseases related to the nervous system, such as stroke, epilepsy, and headache [20]. Gastrodin, as a main active ingredient isolated from *Gastrodia elata* Blume, is a phenolic glycoside compound, possessing various activities, such as antioxidant, anti-inflammatory, vaso-modulatory, and neuroprotective effects against neuronal apoptosis [21]. At the same time, gastrodin exerts anti-AD effects by up-regulating the expression of ChAT in the CA1 region of rat hippocampus and inhibiting the expression of AChE [22]. Furthermore, it reduces oxidative stress and thereby lowers the phosphorylation level of tau protein in the brains of AD model mice, ultimately providing protection for neurons [23].

The close relationship between AD and aging has been proven in various studies. With the increase in life expectancy, Alzheimer’s disease is becoming increasingly common. Regardless of whether Alzheimer’s disease is a normal part of aging or not, the fact that it has such a detrimental impact on people’s lives cannot be ignored any longer [24]. Here, we applied a series of integrated bioinformatics analyses and machine learning models to screen hub aging-related genes in Alzheimer’s disease, which are used to investigate the molecular mechanisms of gastrodin against these genes. We identified 29 intersecting genes among DEGs of AD, key module genes of AD, and aging genes. Next, nine common hub genes were identified by four algorithms of the cytoHubba plug-in. Four hub genes, *GFAP*, *NPY*, *SNAP25*, and *SST*, were found as possibly hub aging-related genes in Alzheimer’s disease from machine algorithms by adopting the random forest, LASSO, SVM, and Boruta models. Among them, *GFAP* and *SNAP25* showed marked upregulation, while NPY and SST exhibited significant downregulation (*p* < 0.05). Finally, molecular docking and molecular dynamics simulation exhibited that gastrodin has an excellent affinity in docking with SNAP25.

In the results of molecular docking, gastrodin showed strong binding affinities with all four targets: GFAP, NPY, SNAP25, and SST, showing binding energies of –4.8, –4.9, –7.5, and –5.9 kcal/mol, respectively. During this process, we only used the protein data from the Protein Data Bank database for molecular docking, without performing a brief molecular dynamics simulation to relax the protein structure in order to expose more docking interaction sites. This was due to considerations regarding the method’s characteristics and computational efficiency. Firstly, we take into account the computational efficiency and cost, as the core advantage of molecular docking lies in its high speed, enabling the rapid screening of large compound libraries. Molecular dynamics simulation is extremely time-consuming and computationally resource-intensive. Even a meaningful MD simulation of just a few nanoseconds could cost much more than thousands of molecular dockings. In this article, our aim is to quickly identify the proteins that have a better compatibility with gastrodin. Introducing MD preprocessing would seriously slow down the entire process and is not cost-effective from a practical perspective. Secondly, the core philosophy of molecular docking is that, based on an experimentally determined, energetically favorable conformation (in the PDB structure), it predicts how the ligand will bind, rather than precisely simulating the dynamic evolution of the protein itself. Furthermore, the X-ray crystal structures or NMR structures in the PDB are regarded as experimentally verified and reliable starting points. The researchers believe that this structure itself is already in a conformation with relatively lower energy. Finally, we considered that a “brief” MD simulation (such as a few nanoseconds) might not be long enough to allow the protein to undergo significant conformational changes and “expose” new sites. It might merely oscillate around the experimental structure.

SNAP25 (Synaptosomal-associated protein, 25kDa) is a crucial synaptic regulatory protein, mainly involved in the release of neurotransmitters and the process of synaptic transmission [25]. It also plays an important role in cardiac electrophysiology and the repair of the neural system. It is worth noting that the loss of synapses in certain brain regions is an early sign of AD, and the loss of synapses occurs before the loss of neurons [26]. Moreover, cerebrospinal fluid SNAP 25 is a newly discovered marker of synaptic damage, and its increase indicates that patients with early AD (i.e., AD-induced SCI) have an elevated ratio of CSF SNAP25 and SNAP25 to Aβ42, while the ratio of CSF SNAP25 and SNAP25 to Aβ42 is very sensitive for the diagnosis of AD. Therefore, CSF SNAP25 can serve as a biomarker for the early diagnosis of AD and can also predict the clinical progression of AD. Interestingly, the ratio of SNAP25, SNAP 25, and Aβ42 is closely related to tau and may serve as an alternative biomarker for targeting tau protein in the future [26,27]. Many studies have shown that CSF SNAP25 increases in the early stage of AD, increases in certain stages of AD, and it has been reported that CSF SNAP25 increases with the severity of AD. This is related to memory loss and a decline in cognitive ability. Interestingly, the level of CSF SNAP 25 decreases longitudinally with the development of AD [28].

However, this study also has some limitations. Although the data mining method provides valuable insights for the research on the AD targets of ginsenosides, validating our conclusions through clinical trials and animal experiments would make them more significant. Therefore, further verification through clinical trials and animal experiments is needed in the future.

## 4. Materials and Methods

### 4.1. Data Preparation

AD gene expression data used in this study (numbers GSE132903 and GSE122063) were collected from the Gene Expression Omnibus (GEO) database (https://www.ncbi.nlm.nih.gov/geo/, accessed on 7 June 2025) (Table 2). The training dataset file (number GSE132903) contained temporal cortex samples of 97 AD patients and 99 healthy controls, annotated using the GPL10558 platform. We also utilized the GSE122063 as the test dataset to validate the hub genes, which included temporal cortex samples from a total of 50 samples (28 AD samples and 22 normal samples) downloaded from the GPL16699 platform. GSE167490 was utilized as a high-throughput single-cell RNA-seq dataset from the temporal cortex of AD patients.

### 4.2. Weighted Gene Co-Expression Network Analysis

WGCNA was considered a bioinformatics analytical method to identify key gene modules associated with AD. The WGCNA tool in R was used to construct a co-expression network by filtering low-variance genes and obtaining transcriptomic data from both control and AD groups. Next, the PickSoftThreshold function was used from the WGCNA package 1.73 to select and confirm the appropriate soft threshold β. An adjacency matrix was transformed from the matrix data in this process and then clustered. After determining the module eigengenes (MEs) and combining similar modules in the tree according to ME, we constructed the clustering dendrograms. The hub genes in the target module were determined through an integrated analysis of module membership (MM) and gene significance (GS) metrics.

### 4.3. Data Preprocessing and Differential Expression Genes Screening

Differential expression genes (DEGs) between the normal group and AD group were identified by using the limma R tool for GSE132903 after data was normalized and preprocessed for pairwise comparisons. DEGs were identified with thresholds of *p*-value < 0.05 and |log2FC| > 0.585.

### 4.4. Identification of Aging-Related Differential Expression Genes

A total of 1,985 aging genes were obtained from the public databases Human Aging Genomic Resources (https://genomics.senescence.info/cells/, accessed on 15 June 2025), GeneCards databases (https://www.genecards.org/, accessed on 15 June 2025), and from the literature (Supplementary Table S1). To systematically identify aging-related differentially expressed genes, we performed intersection analysis of three distinct gene sets: the DEGs, genes from key modules (WGCNA), and aging genes.

### 4.5. Functional Enrichment Analysis

The biological processes (BP), cellular components (CC), and molecular functions (MF) of Gene Ontology (GO) analysis and KEGG pathway analysis were performed using the Sangerbox 3.0, a free online platform for data analysis (http://www.sangerbox.com/tool.html, accessed on 15 June 2025). The “GSVA” R package 2.2.0 was employed for calculating the process score by the gene set variation analysis (GSVA) algorithm. Meanwhile, Gene set enrichment analysis (GSEA) was performed provide insights into significantly differential functions between control and AD groups.

### 4.6. PPI Network Analysis

The protein–protein interaction (PPI) networks of the identification genes were analyzed by the STRING tool (https://string-db.org/). Cytoscape 3.7.0 was employed to visualize the most closely related gene clusters by the MCODE plug-in and indicate the lines between nodes in the PPI network. The top 10 targets according to the BC, CC, and DC values were determined in various CytoHubba modules, including EPC, MCC, MNC, and Degre. Thus, we identified the common hub genes from the results of four algorithms.

### 4.7. Machine Learning and Clinical Characteristic Analysis

To identify hub aging-related genes in Alzheimer’s disease, a series of advanced machine learning algorithms were employed, which encompassed support vector machines (SVM), least absolute shrinkage and selection operator (LASSO) regression, random forest (RF), and Boruta. Among them, we use the SVM algorithm for regression and classification tasks. LASSO is a regression analysis algorithm executed using the “glmnet” package 4.1.10 to identify genes connected with binary classification outcomes. Using the “randomForest” package 4.7.1.2 in R, variable importance and key genes could be assessed and selected by executing the RF algorithm. The Boruta is a machine learning method that could identify the truly important features and distinguish the irrelevant ones. Receiver operating characteristic (ROC) curves were constructed to assess the diagnostic potential and robustness of the identified core targets by using the “pROC” package 1.18.5 in R and computing the area under the ROC curve (AUC).

### 4.8. Assessment of Immune Cell Infiltration

The infiltration scores of immune-related cells between AD patients and healthy controls were analyzed through the CIBERSORT algorithm to assess the relative proportions of 22 lymphocyte subtypes in each sample group. Subsequently, we used the Spearman correlation analysis to evaluate the relationship between core target genes and immune cell infiltration.

### 4.9. Single-Cell Analysis and Subcellular Localization

We analyzed the AD single-cell dataset, GSE167490, utilizing the “Seurat” package 5.3.0 and “SingleR” package 2.10.0 in R software. To ensure robust cellular data quality, we applied a stringent filtering criterion by selecting only genes detected in a minimum of three individual cells. To analyze the changes in biomarkers in the disease process, the COMPARTMENTS database (https://compartments.jensenlab.org/, accessed on 15 June 2025) was employed to visualize the subcellular localization of the biomarker proteins.

### 4.10. Molecular Docking and Molecular Dynamics Simulations

We obtained the 3D structures of the hub aging-related genes in AD from the Protein Data Bank (http://www.rcsb.org/), and the molecular structure of gastrodin was downloaded from the PubChem database (http://pubchem.ncbi.nlm.nih.gov/). We use ChemDraw 17.1 to draw the structure formula of gastrodin for docking and pour it into Chembio3D 17.1 for energy minimization, and then import AutodockTools-1.5.6 to add hydrogen, calculate the charge, assign charge, set rotatable keys, and save it as “pdbqt”. After dehydration and hydrogenation of the proteins, we use AutoDockTools (version 1.5.7) for molecular docking to comprehensively evaluate the conformation of the ligand and its interaction with the binding pocket of the protein. The docking results are sorted by score, and the conformation with the highest score is selected as the optimal pose. The key protein structures exhibiting the highest binding affinities were selected for visualization and analyzed using PyMOL version 3.2.

Molecular dynamics simulations were conducted using the GROMACS (version 2020.6) [29]. Water molecules were modeled as a simple point charge model (SPC216). The entire system was neutralized by incorporating either four sodium ions (at concentrations of G719S and T790 M) or three sodium ions (at concentrations of L858R and T790 M/L858R). The GROMOS96 43a1 force field was applied to determine chemical interactions. Energy minimization of the system was achieved through the steepest descent followed by the conjugate gradient algorithms. The next step involved a “position restraint procedure,” which was performed in conjunction with NVT (constant volume and temperature) and NPT (constant pressure and temperature) ensembles. An NVT ensemble was maintained at 300 K with a coupling constant of 0.1 ps for a duration of 1000 ps. The NPT ensemble was conducted with a constant pressure of 1 bar and a coupling constant of 5.0 ps for 1000 ps. Pressure was kept constant at 1 bar using the Parrinello-Rahman barostat [30]. The LINCS algorithm was utilized to enforce covalent bond constraints. To manage long-range electrostatic and van der Waals interactions, the Particle Mesh Ewald (PME) method and cut-off techniques were employed, respectively [31]. During the molecular dynamics (MD) simulations, the stability of energy, temperature, and density of the system was evaluated. Several criteria were monitored to assess stability, including the root-mean-squared deviations (RMSDs) of the backbone atoms, the root mean square fluctuations (RMSF) of the Cα atoms, the radius of gyration (Rg) of the backbone atoms, as well as intramolecular and intermolecular hydrogen bonds, and the solvent accessible surface area (SASA) of both the ligand and protein. All simulations were conducted over a duration of 100 ns for each ligand–protein system under investigation, with an integration step of 2 fs.

### 4.11. Statistical Analyses

All data processing and analysis were conducted using R software (version 4.5.0) unless otherwise stated. *p* < 0.05 was considered statistically significant, and the significance level is denoted as follows: * (*p* < 0.05), ** (*p* < 0.01), and *** (*p* < 0.001) by one-way ANOVA.

## 5. Conclusions

Based on WGCNA, differential gene expression and aging genes, we used protein–protein interaction (PPI) network and machine learning to screen four signature aging-related AD genes, namely *GFAP*, *NPY*, *SNAP25*, and *SST*. In the validation of the training dataset and test dataset, *GFAP* showed marked upregulation, while *NPY*, *SNAP25*, and *SST* exhibited significant downregulation (*p* < 0.05). Next, immune cell infiltration in AD patients and its correlation with the above four genes were evaluated, offering a new perspective on he changes in the immune environment of AD patients. Furthermore, the study on the expression and subcellular localization of GFAP, NPY, SNAP25, and SST in temporal cortex tissue has enabled us to gain a deeper understanding of the roles of GFAP, NPY, SNAP25, and SST in AD. Finally, we found that gastrodin has an excellent affinity in docking with SNAP25 based on molecular docking and molecular dynamics simulation (–7.5 kcal/mol), which is much higher than that of other receptors (GFAP: –4.8 kcal/mol, NPY: –4.9 kcal/mol, SNAP25, and SST: –5.9 kcal/mol). The binding affinity reflects the possibility of protein receptors binding to small molecule ligands. The complex formed by gastrodin and SNAP25 has a low binding energy and a high binding affinity, and its conformation is relatively stable. However, this study also has some limitations. The result could expand the sample size and conduct in-depth research in future in vivo and in vitro experiments. Encouragingly, we have successfully obtained aging-related biomarkers and explained the potential therapeutic ability of gastrodin in SNAP25. These findings provide valuable insights into the application and further development of gastrodin in anti-aging and Alzheimer’s disease drugs.

## Figures and Tables

**Table 1:** The hub genes.

Gene Symbol	Gene Name	Score of Degree	Score of EPC	Score of MCC	Score of MNC
*GFAP*	Glial fibrillary acidic protein	11	9.38	52	9
*SNAP25*	Synaptosomal-associated protein 25	10	9.28	56	10
*NFKBIA*	NF-kappa-B inhibitor alpha	6	7.28	6	2
*UCHL1*	Ubiquitin carboxyl-terminal hydrolase isozyme L1	6	8.02	15	5
*CCK*	Cholecystokinin receptor type A	5	8.02	25	4
*SST*	Serine O-succinyltransferase	5	8.04	30	5
*NPY*	Pro-neuropeptide Y	5	8.23	30	5
*GABRG2*	Gamma-aminobutyric acid receptor subunit gamma-2	5	7.88	18	5
*SNCA*	α-Synuclein	5	7.30	10	5
*GABRA1*	Gamma-aminobutyric acid receptor subunit alpha-1	4	7.33	12	4

**Table 2:** Details of the GEO datasets.

Dataset	Platform	Number of Samples (Groups)
GSE132903	GPL10558 Illumina HumanHT-12 V4.0 expression beadchip	196 (AD 97; Normal 99)
GSE122063	GPL16699 Agilent-039494 SurePrint G3 Human GE v2 8x60K Microarray 039381 (Feature Number version)	50 (AD 28; Normal 22)
GSE167490	GPL24676 Illumina NovaSeq 6000 (Homo sapiens)	10 (AD 10)

## Data Availability

The data will be available upon request.

## Supplementary Materials

The following supporting information can be downloaded at: https://www.mdpi.com/article/10.3390/ijms26189097/s1.

## Abbreviations

The following abbreviations are used in this manuscript:

GEO	Gene Expression Omnibus
NCBI	National Center for Biotechnology Information
WGCNA	Weighted gene co-expression network analysis
DEGs	Differentially expressed genes
GO	Gene Ontology
KEGG	Kyoto Encyclopedia of Genes and Genomes
PPI	Protein–protein interactions
GSVA	Gene set variation analysis
GSEA	Gene set enrichment analysis
SVM	Support vector machines
LASSO	Least absolute shrinkage and selection operator
RF	Random forest
ROC	Receiver operating characteristic
